# Advances in Novel Nanomaterial-Based Optical Fiber Biosensors—A Review

**DOI:** 10.3390/bios12100843

**Published:** 2022-10-08

**Authors:** Muyang Li, Ragini Singh, Yiran Wang, Carlos Marques, Bingyuan Zhang, Santosh Kumar

**Affiliations:** 1 Shandong Key Laboratory of Optical Communication Science and Technology, School of Physics Science and Information Technology, Liaocheng University, Liaocheng 252059, China; 2 College of Agronomy, Liaocheng University, Liaocheng 252059, China; 3 Department of Physics & I3N, University of Aveiro, 3810-193 Aveiro, Portugal

**Keywords:** biosensors, nanomaterials, optical fiber biosensor, sensitivity, surface plasmon resonance

## Abstract

This article presents a concise summary of current advancements in novel nanomaterial-based optical fiber biosensors. The beneficial optical and biological properties of nanomaterials, such as nanoparticle size-dependent signal amplification, plasmon resonance, and charge-transfer capabilities, are widely used in biosensing applications. Due to the biocompatibility and bioreceptor combination, the nanomaterials enhance the sensitivity, limit of detection, specificity, and response time of sensing probes, as well as the signal-to-noise ratio of fiber optic biosensing platforms. This has established a practical method for improving the performance of fiber optic biosensors. With the aforementioned outstanding nanomaterial properties, the development of fiber optic biosensors has been efficiently promoted. This paper reviews the application of numerous novel nanomaterials in the field of optical fiber biosensing and provides a brief explanation of the fiber sensing mechanism.

## 1. Introduction

Researchers have recently preferred fiber optic biosensors because of the benefits of their high sensitivity, excellent stability, good specificity, and cheap analytical cost [1,2,3,4]. In terms of price, size, and convenience of in situ label-free technique development, the use of fiber optic devices as biological/chemical sensing components provides numerous important benefits over electrochemical approaches. As a result, fiber-based biosensors have gained popularity and provide a novel approach for the wide range detection of biological analytes [5,6,7,8]. Many geometrically modified and microstructured fibers, including side-polished fibers [9,10], D-shaped fibers [11,12,13,14], U-shaped fibers [15], tapered fibers [16,17,18,19], microsphere structured fibers [20,21], photonic crystal fibers (PCF) [22,23,24,25], and grating fibers [26,27,28,29,30], are employed to draw the guiding mode out of the cladding to interact with the surrounding environment. In order to further increase the sensitivity in the detection of bio-analytes at low concentrations, long period gratings (LPG) [31,32] and tilted fiber gratings (TFG) [28,33,34] are frequently used in biosensors. These gratings achieve surface plasmon resonance (SPR) effects and selective or enzymatic coatings, respectively. For sensitive and precise detection of bacterial flora and biomolecules, optical biosensors based on fluorescence [35,36,37,38,39], SPR [3,5,13,40,41], and localized surface plasmon resonance (LSPR) [7,18,42,43] are currently being reported.

Fiber optic biosensors offer a cheap, accurate, and label-free detection method for bioanalysis, marine life, and medical diagnosis [44,45,46]. Due to their superior electrical, magnetic, thermal, and optical capabilities, nanomaterials have shown tremendous development in several domains [47,48] by combining fiber optic biosensors with nanomaterials. Due to their tiny size impact, surface effects, quantum size effects, and macroscopic quantum tunneling effects, nanomaterials display peculiar chemical and physical characteristics. Nanoparticles exhibit several benefits due to facile surface modification, controlled size, and straightforward characterization to meet the versatility and miniaturization requirements of sensors. The development of sensors also depends on additional requirements, including strong selectivity, broad detection range, and high sensitivity [49,50,51]. The aforementioned criteria can be easily met by nanomaterials. Thus, nanomaterials have improved the detection capabilities of biosensors and encouraged the development of novel biosensors.

In the present era, nanomaterials cover almost every field with diverse applications as they play a crucial role in science and technology. There are many applications of nanomaterials in the medical field, food industry, agriculture, and commercial industry [52,53]. Nanomaterials have attracted great interest from the scientific community due to their unique microstructure and unusual properties and are now applied in booming sensing research fields, including biology, chemistry, and physics [47,51,54,55]. Quantum dots (QDs), metal nanoparticles, metal clusters, and other formations are examples of zero-dimensional (0D) nanomaterials that enhance nanostructures and take the form of QDs [35,38,48]. The representative types of one-dimensional (1D) nanomaterials include nanowires, nanotubes, and nanorods that are widely used in the field of biosensors. As a type favored by researchers, two-dimensional (2D) nanomaterials have more extensive applications. These nanomaterials include graphene oxide (GO), molybdenum disulfide (MoS_2_), MXene, metal films, and other nanomaterials with good optical properties and biocompatibility. Three-dimensional porous nanomaterials with interconnected pores can realize multi-channel analyte diffusion, which enhances the uniform and dense adsorption of plasma nanostructures to biological substances, so as to effectively identify the object to be tested. They have shown great momentum in several research disciplines, including biological imaging and biosensors, as a result of their exceptional performance.

**Figure 1:**
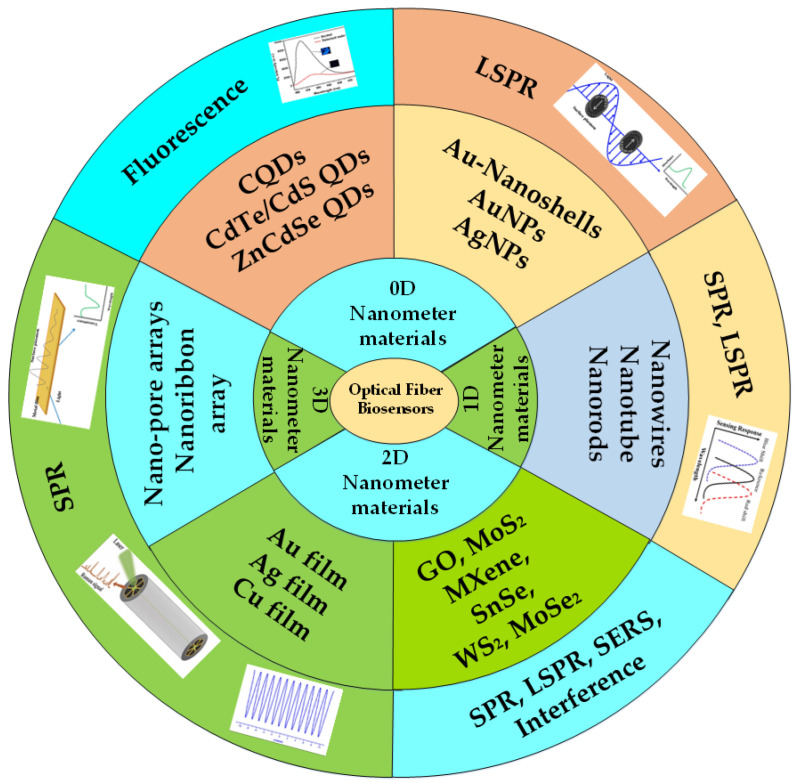
Schematic representation of the novel nanomaterials used in different sensing applications.

Until now, many researchers have used various nanomaterials in the development of fiber optic biosensors based on different sensing principles. Optical fiber biosensors based on nanomaterials are the future platform for rapid and cost-effective biomarker detection. The application mechanism of different nanomaterials is also different. All kinds of QDs in 0D nanomaterials are mainly used in the design of fluorescent biosensors. However, metal nanoparticles and nanoshells in 0D nanomaterials are necessary conditions for LSPR effect excitation. Nanowires, nanotubes, and nanorods in 1D nanomaterials have been applied to the research of SPR and LSPR biosensors due to their excellent dielectric effects and optical properties. The electron transport efficiency, surface area, and biocompatibility of 2D nanomaterials are greatly improved, which enhances the performance of fiber optic biosensors. Additionally, 0D and 1D nanomaterials are different from 2D nanomaterials where the atom is exposed to most of their surface, leading to the display of unique physical and chemical properties and unique surface chemistry, especially those biodegradable nanomaterials that may be promising for biological sensing applications of nanomaterials. Therefore, many researchers are gradually becoming devoted to the application of 2D nanomaterials in the field of optical fiber biosensing based on SPR, LSPR, and the surface-enhanced Raman scattering (SERS) effect. Two-dimensional metal films and three-dimensional (3D) nanoarrays play an important role in enhancing the local electric field of optical fiber structures so they can be used in the development of SPR-based optical fiber biosensors. These sensing systems can detect a large number of molecules of clinical relevance, including glucose [56,57], bacteria [58,59], cancer cells [6,60], dopamine [61,62,63], deoxyribonucleic acid (DNA) [41], and so on. The applications of different types of nanomaterials based on different sensing principles are represented in Figure 1.

**Figure 2:**
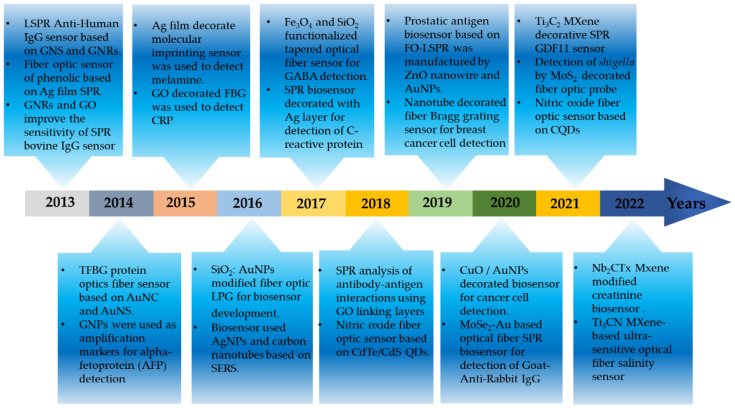
Outstanding progress of novel nanomaterials in the field of fiber optic biosensors in the past decade.

In the last decade, many studies related to nanomaterial-modified fiber sensing probes have been published. The initial researchers mainly relied on GO, a metal film-modified fiber sensing probe whose sensing performance is limited by the synthesis and application of immature nanomaterials. With the rapid development of the application of nanomaterials in recent years, the original application of nanomaterials has become more mature. In addition, new nanomaterials such as MoS_2_, MXene, metal oxides, and non-metal oxides are gradually entering the field of optical fiber biosensors. These materials have better optical properties and biocompatibility, which greatly improves biosensing performance and allows for the detection of some viruses, bacteria, cancer cells, and other substances. Figure 2 shows the year-wise application of fiber optic biosensors in the detection of several analytes. Therefore, this review paper introduces the outstanding achievements of nanomaterials in the field of fiber sensing in recent years, comprehensively compares the classification and application of nanomaterials in different dimensions, and comprehensively introduces the interaction of nanomaterials in these recognition systems.

## 2. Application of 0D Nanomaterials in the Field of Optical Fiber Biosensors

The 0D nanomaterials widely used in the field of optical fiber biosensing include various kinds of QDs, metal nanoparticles, and nanoshells. Among these, QDs are mainly used in the research of biosensors due to their fluorescence luminescence or fluorescence resonance properties, whereas metal nanoparticles and nanoshells are mainly used based on their LSPR property. Zero-dimensional nanomaterials mostly exist in spherical or quasi-spherical shapes with diameters less than 100 nm [47,64]. The most common 0D materials used in sensing applications are carbon QDs (CQDs) [65,66], semiconductor quantum dots (SQDs) [67,68], graphene quantum dots (GQDs) [62,69,70,71], CdTe QDs [72,73,74], and precious metal NPs such as silver nanoparticles (AgNPs) and AuNPs [18,20,75,76,77,78]. However, 0D nanomaterial-based sensing is still in its infancy, and the practical applicability of these nanosensors still has room for advancement, which needs to be solved shortly.

### 2.1. Quantum Dots

**Figure 3:**
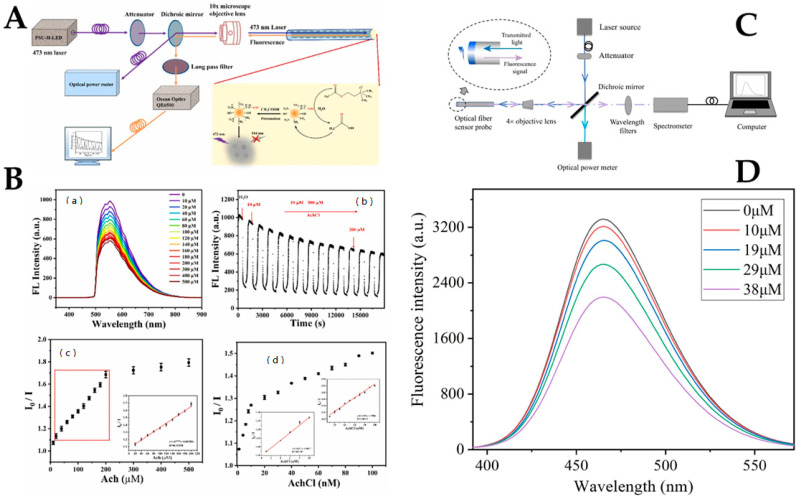
(**A**) Schematic diagram of optical fiber fluorescence experiment platform. (**B**)(a), The fluorescence spectra of the optical fiber sensor in the presence of AchCl solution with different concentrations ranging from 10 to 500 μM; (**B**)(b), The AchCl detection time sequence diagram; (**B**)(c), The calibration curve of the optical fiber sensor for the determination of AchCl ranging from 10 to 500 μM; and (**B**)(d), The calibration curve of the optical fiber sensor for the determination of AchCl ranging from 1–100 nM. Reprinted with permission from Sensors and Actuators B: Chemical, Copyright 2019, Elsevier [66]. (**C**) Experimental setup of NO fiber optic biosensor. (**D**) Fluorescence intensity of CQDs after adding different concentrations of NO. Reprinted with permission from Journal of Photochemistry and Photobiology A: Chemistry, Copyright 2019, Elsevier [73].

As a novel nanomaterial, CQDs are widely used in chemical sensors [36,79], biosensors [56,65,66], and biomedical applications [80,81] due to their properties, which include low toxicity, high biocompatibility, and high chemical stability [38,82]. QDs can stably immobilize biological receptors on the surface of QDs through the exchange of chemical bonds with ligands, which ensures the sensors have good specificity, which is a prerequisite to achieving sensitivity to target antibodies and specific sensor response. [83,84]. Zhang et al. reported a novel acetylcholine fiber optic biosensor based on a nitrogen-doped fluorescent CQDs/acetylcholinesterase (N-CQDs/AchE) compound. The developed sensor determined the relationship between the fluorescence intensity of the acetylcholine chloride solution and the N-CQDs/AchE complex [66]. The optical fiber fluorescence experimental platform for Ach detection is shown in Figure 3A. The mechanism of Ach detection was the generation of acetic acid hydrolyzed by Ach and AchE to fluorescence quench N-CQDs. The concentration of Ach was detected by the change of fluorescence intensity because there was a dependence between the concentration of Ach and the quenching degree of the fluorescence intensity. As shown in Figure 3B(a), an increase in acetylcholine chloride (AchCl) concentration resulted in a decrease in fluorescence intensity because acetic acid produced by AchE hydrolysis reduced the pH of the system. The real-time fluorescence response of the optical fiber sensor to AchCl was tested in the range of 10–500 μM, as shown in Figure 3B(b). As a result, when the fiber probe was immersed in the test solution, the fluorescence intensity decreased significantly within 1 min and then gradually stabilized within 5 min. The fluorescence intensity of the probe decreased to some extent before reaching full stability because it took some time for AchCl to diffuse into the cellulose acetate membrane to neutralize the progression of the enzymatic reaction. From Figure 3B(c), the linear range was determined to be 20–200 μM, and the limit of detection (LoD) was 16.28 μM. Figure 3B(d) shows that the range of the fluorescence intensity ratio was linearly dependent on AchCl concentration. In conclusion, the AchCls fiber optic sensor had the highest sensitivity in the range of 1–10 nM. The hydrophilicity and stability of N-CQDs in aqueous systems may be enhanced by adding groups such as amino and hydroxyl groups. The approach described in this study, which simply involved immobilizing AchE, is likely to accomplish real-time online detection of acetylcholine since it is simpler, smaller, and has no electromagnetic interference. The fluorescent fiber biosensor based on the enzymatic reaction of acetic acid to reduce the environmental pH value and quench the carbon point has the advantages of fast response, high sensitivity, and wide detection range.

Wu et al. designed a fiber optic biosensor probe based on CQDs for nitric oxide (NO) detection in serum using the combined beneficial effect of fiber optic sensing and fluorescence analysis [73]. The optical fiber NO sensing system was designed and constructed based on the principle of optical fiber sensing, as shown in Figure 3C. The o-phenylenediamine group on the surface of the CQDs reacts with NO to generate an electron-free triazole structure, which is then detected by the sensor. The static quenching phenomenon is caused by the formation of a non-luminescent ground state complex between the fluorescent substance and the quencher. The ground state complex competes with the ground state molecules of the fluorescent substance to absorb the excitation light, thereby reducing the fluorescence intensity of the fluorescent substance. The change in fluorescence of the CQDs with various NO concentrations ranging from 10 μM to 38 μM were measured in an aqueous solution, and the results are shown in Figure 3D. With the increase in NO concentration, the fluorescence intensity of the CQDs gradually decreased, indicating that the CQDs had a good response to NO. The developed biosensor was able to detect NO levels in aqueous media. The linear detection range of NO was from 1 × 10^−8^ to 1 × 10^−4^ mol/L with an LoD of 9.12 nM. The NO fiber biosensor verifies the feasibility and reliability of the proposed fiber biosensor for NO detection, which is expected to promote the development of NO detection and provide more help for the detection and prevention of some clinical diseases. However, in practical applications, the detection environment is more complex, and the preparation of sensitive film and optical sensing platforms should be optimized to further improve the performance of the sensor.

Although great progress has been made in QDs-based biosensors, their application in fiber optic biosensors is still at an immature level due to several reasons, including the potential toxicity of QDs, which limits their real-time application in biological imaging and biomedicine. Thus, improving the stability of QDs in complex environments is a necessary condition for constructing safe and environmentally friendly biosensors. The majority of QDs surface markers are based on streptavidin-biotin interactions and N-hydroxysuccinimide (NHS)/ethyl (Dimethylaminopropyl) carbodiimide (EDC) coupling reactions, which may include non-specific adsorption, drawn-out processes, and washing stages. To make labeling schemes simpler, increase labeling effectiveness, and reduce undesired adsorption, more straightforward and efficient QDs surface labeling techniques are urgently required. Biosensors based on QDs are usually employed in the detection of biological molecules. However, biosensors with a simultaneous detection ability for multiple biological compounds are desired, which can be achieved using QDs of different colors or developing multiple fluorescence biosensors based on a single QDs [85,86]. With these initiatives, fiber optic biosensors will soon significantly advance fundamental research, drug development, and clinical diagnostics. Table 1 shows some research achievements of QDs in the field of optical fiber biosensors in recent years.

### 2.2. Metal Nanoparticles

Noble metal nanoparticles have remarkable plasma, catalytic, and electronic properties [89,90,91]. They can interact with a variety of biomolecules, such as enzymes, oligonucleotides, aptamers, and antibodies [18,75,91,92,93]. Due to a lack of biological activity, nanomaterials like AuNPs can be used to immobilize proteins without altering their integrity. AuNPs, therefore, are widely used in the detection of several biological molecules and are the most commonly used nanoscale markers for biosensors due to several advantages, such as excellent photoelectric properties, low toxicity, and easy and low-cost synthesis [94,95,96]. AuNPs can amplify electrochemical and optical signals intensively [97,98,99] and are often used as an enlargement label. Biomarkers have been immobilized by simple amine conjugation and avidin-biotin fixation. In addition, AuNPs also have unique LSPR properties with a high molar extinction coefficient due to their size variations in the presence of the target analyte [100,101]. For example, colloidal suspensions of AuNPs with a diameter of 10 nm take on the color of red wine and produce an LSPR peak at 520 nm in the UV-visible absorption spectra. When the receptors on the surface of AuNPs react with the object to be measured, the refractive index (RI) of the medium around the AuNPs changes, and the LSPR resonance peak experiences redshift [18,57,76]. The extent of the redshift depends on the change in the RI of the surrounding medium. Kumar et al. developed a biosensor probe based on LSPR for the detection of biological substances and microbes, including cholesterol [18,75], uric acid (UA) [20,102], and *Shigella* Bacteria [103].

**Figure 4:**
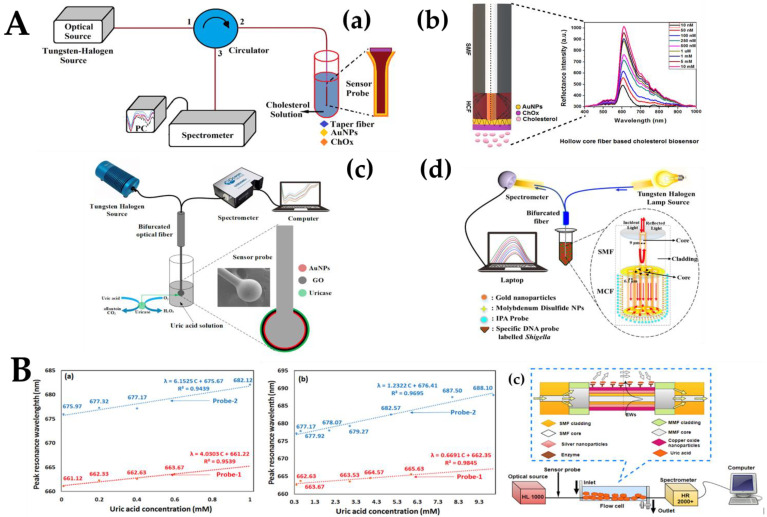
(**A**)(a) Experimental setup for tapered fiber biosensor probe. Reprinted with permission from Biomedical Optics Express, Copyright 2019, Optica [18]. (**A**)(b) Hollow-core fiber-based cholesterol biosensor. Reprinted with permission from IEEE Sensors Journal, Copyright 2019, IEEE [75]; (**A**)(c) Micro-ball fiber structure-based experimental setup. Reprinted with permission from IEEE Transactions on NanoBioscience, Copyright 2020, IEEE [20]. (**A**)(d) Experimental setup for biosensor detection of *Shigella* bacteria. Reprinted with permission from Journal of Lightwave Technology, Copyright 2020, IEEE [103].. Linearity plot of the probes: analysis of sensing probes for the detection of UA presents in (**B**)(a) serum and (**B**)(b) urine; (**B**)(c), Experimental setup for the detection of UA. Reprinted with permission from IEEE Transactions on Instrumentation and Measurement, Copyright 2020, IEEE [102].

The tapered single-mode fiber (SMF) transmittance sensing probe based on cholesterol oxidase (ChOx) functionalization is shown in Figure 4A(a). The probe structure was a tapered fiber structure, with a waist diameter of about 4 μm and a waist length of 10 mm. The light source operated at the visible and near-infrared spectrums. The AuNPs fixed tapered fiber optic probe was attached to the circulator’s second port [18]. On the surface of the tapered fiber, the LSPR phenomenon occurred, and the surface area was increased due to the small size of AuNPs ChOx enzyme hydrolyzed the cholesterol and catalyzed the breakdown of cholesterol into H_2_O_2_ and cholestenone in the presence of oxygen. The level of H_2_O_2_ changed the RI of the AuNPs surface and reflection intensity. Figure 4A(b) describes a cholesterol biosensor using SMF and hollow-core fiber (HCF) with a hetero-core structure for detecting and measuring cholesterol concentration in the human body [75]. SMF was used for the transmission of optical signals, and the HCF was used as a sensing probe for the detection of biological substances. The amino conjugation fixation technique was used to permanently attach 11 ± 0.5 nm AuNPs to the probe’s surface. ChOx enzyme functionalization over the sensor probe further improved the biosensor’s selectivity. Due to the LSPR phenomenon, the SMF-HCF sensor probe’s sensitivity was highly enhanced. Further, Figure 4A(c) shows a highly sensitive and selective spherical fiber optic enzyme biosensor for the detection of UA in human serum. An SMF sensor with a 350 μm diameter probe was fabricated using a special fiber fusion mechanism (FSM-100P+ ARC Master, Fujikura, Japan) [20]. AuNPs and GO were coated onto the surface of the fiber probe to stimulate the LSPR phenomenon and increase the biocompatibility of the sensor probe, respectively. Thereafter, uricase was functionalized over the spherical sensor probe to ensure specificity and selectivity. Fungus dysentery is a common and frequently occurring disease in the world, with a high incidence among children and young adults. Figure 4A(d) shows a biosensor based on LSPR composed of a multicore fiber MCF) and SMF splicing for the detection of *Shigella* bacteria [103]. The evanescence waves (EWs) and mode coupling between MCF cores were improved by controlling the etching process. The suggested sensor’s sensitivity to change in the RI was additionally enhanced by the etching procedure. Nanomaterials, including AuNPs and molybdenum disulfide (MoS_2_), were also used to make local plasma excitation easier. Here, oligonucleotide probes specific for *Shigella* were used as recognition elements. The detection and measurement of *Shigella* with the developed sensor were quick and accurate.

AgNPs, similar to AuNPs, are noble metal nanoparticles often used in the detection of metabolites and have size-dependent optical, color, and sensing behaviors owing to LSPR [61,77,104]. AgNPs are more cost-effective and electrically active and have a higher extinction coefficient. However, due to their low stability, AgNPs have not been widely used in the field of fiber optic biosensors. Mach–Zehnder interferometer (MZI) fibers based on SMF-MMF-SMF-MMF-SMF (SMSMS) with copper oxide nanoparticles (CuO-NPs) and AgNPs-based LSPR sensor were demonstrated by Agrawal et al. [102]. This study presented LSPR-based technology with high biocompatibility and sensitivity by using AgNPs and CuO-NPs. CuO and AgNPs/CuO were impregnated on the surface of the SMSMS fiber structure to make the sensing probes. The sensing probe modified with CuO was named Probe-1, and the sensing probe modified with AgNPs/CuO was named Probe-2. In the fabricated sensors, the LSPR phenomenon mainly occurred in the central SMF part of the proposed SMSMS structure. The experimental setup shown in Figure 4B(c) was used to conduct a comparative study on the performance of the proposed sensors. The spectrum between the resonance wavelength and the normalized intensity was recorded for a wide range of UA samples in serum and urine, respectively. Figure 4B(a,b) show the linear correlation of UA detection in serum and urine, respectively. Figure 4B(a,b) also shows the sensitivity, correlation coefficient, and LoD of Probe-1 and Probe-2 in serum and urine, respectively. The sensitivities of the two probes in serum were 4.03 nm/mM and 6.15 nm/mM, respectively. The sensitivities of the two probes in urine were 0.67 nm/mM and 1.23 nm/mM, respectively. These results prove that AgNPs can enhance the LSPR effect and improve the sensing performance. There are many benefits of the developed sensor, such as its excellent selectivity, reusability, repeatability, and lowest LoD in the detection of UA in serum and urine. The uricase functionalized LSPR sensor with a modified SMSMS structure with AgNPs and CuO-NPs had good performance compatibility and excellent application prospects. However, the instability of AgNPs and the high loss of SMSMS are the drawbacks of the probe.

In recent years, nanomaterials such as metal nanoparticles have attracted extensive attention in biosensor applications due to their high surface area, tunable optical properties, surface modification ability, size-dependent enhanced efficiency and specificity, and surface modification ability. Au can form a strong Au-S covalent bond with the thiol group. AuNPs can be well combined with nanotechnology and biological detection technology to achieve single or multiple labeling, which has been widely used in many fields of medicine and biology. AuNPs are also less eco-toxic and more biocompatible than AgNPs [105,106]. Therefore, researchers are more interested in target-specific biosensors using AuNPs. Table 2 lists some AuNPs applications in the field of fiber optic biosensors.

### 2.3. Other 0D Nanomaterials

This section describes the noble 0D nanomaterials that have not yet been widely used in fiber optic biosensors. In addition to Au and Ag nanoparticles, Cu nanoparticles have also been investigated by researchers for their application in optical sensors. Among them, copper oxide (CuO) serves as an excellent semiconductor oxide and provides an effective platform for the immobilization of sensing enzymes [117]. CuO has unique optical properties and the ability to adsorb biological molecules [118]. Singh et al. fabricated an LSPR cell sensing probe based on a CuO nanoflower modification for the efficient detection of cancer cells and achieved remarkable results [60].

**Figure 5:**
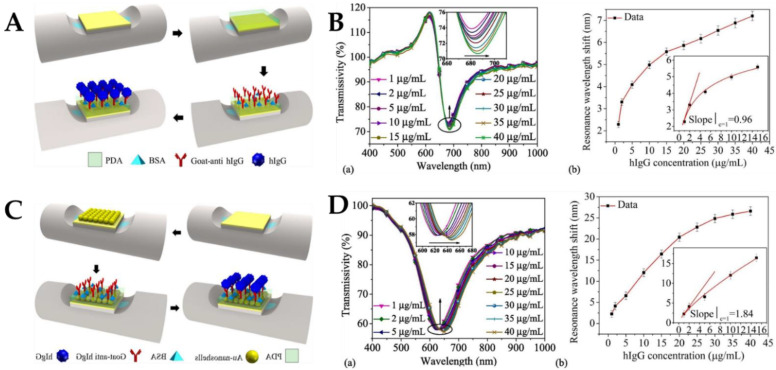
(**A**) Process of polydopamine modification, antibody immobilization, and antigen detection. (**B**) Experimental results of hIgG detection using the LRSPR sensor. (**C**) Process of Au nanoshell modification, antibody immobilization, and antigen detection. (**D**) Experimental results of hIgG detection using the Au nanoshell modified LRSPR sensor. Reprinted with permission from Optics & Laser Technology, Copyright 2021, Elsevier [121].

Similar to AuNPs, Au nanoshells have unique optical and electrical properties with excellent biocompatibility. More binding sites with target molecules can be increased to enhance the absorption of target molecules and improve the sensitivity of the sensor [119,120]. Zhan et al. developed a long-range SPR (LRSPR) biosensor to detect human immunoglobulin G (hIgG) by the surface modification of optical fibers with Au nanoshells [121]. LRSPR can efficiently identify biological macromolecules because of its deep penetration ability and extended propagation distance. Dopamine chemical attachment has good hydrolysis resistance, so dopamine is used for immobilized antibodies. Figure 5A depicts the sensing structure of the side-polished fiber LRSPR biosensor and the specific process of sensor manufacturing. When hIgG and goat anti-hIgG are specifically combined, the RI of the sensor surface changes, and the characteristics of the transmitted light change. The detection of hIgG was achieved by detecting the motion of the resonant dip in the resonant spectrum. Figure 5B(a) shows that the resonance wavelength was redshifted from 2.26–7.20 nm when hIgG concentration was changed from 1–40 μg/mL. Figure 5B(b) shows that the sensitivity of the sensor was 0.96 nm/(μg/mL), and the LoD was 0.38 μg/mL. As a result of the plasma coupling effect between Au nanoshells and Au films, the electric field intensity at the sensor’s surface may be increased, allowing for a lower LoD and greater sensitivity with the suggested sensor construction. The improved LRSPR sensor made from Au nanoshells was quite useful in real-life situations. The biosensor’s portability, sensitivity, and detection range make it a useful tool for biochemical sensing applications. Mercaptan chemical bonding is an effective way of affixing the Au nanoshells to the SPR. The SPF-LRSPR biosensor modified with Au nanoshells was used for hIgG detection. Figure 5C depicts the sensing structure of the side-polished fiber LRSPR biosensor modified by Au nanoshells and the specific process of sensor fabrication. The experimental results are shown in Figure 5D. Figure 5D(a) shows that when hIgG concentration changes from 1–40 μg/mL, the resonance wavelength redshifts from 2.29–26.61 nm. According to Figure 5D(b), the sensitivity was 1.84 nm/(μg/mL), and the LoD was 0.20 μg/mL. The modified Au nanoshells produced more scattering on the surface of the sensor, making the resonance spectrum of the sensor wider than that of the unmodified sensor.

## 3. Application of 1D Nanomaterials in the Field of Optical Fiber Biosensors

One-dimensional nanomaterials with good surface chemical properties and biocompatibility, such as nanotubes, nanorods, and nanowires, are mainly used in fiber optic biosensors based on SPR and LSPR principles. Their excellent optical properties and biocompatibility can effectively improve the sensitivity of biosensors and reduce LoD.

### 3.1. Nanowires

Recently, due to their extreme sensitivity, cheap cost, and low power consumption, nanowire-integrated biosensors are considered a top sensing platform for biomarker detection [122]. In comparison to other nanostructures, 1D nanowires provide a number of benefits for molecular sensing that can be summed up as improved sensing capability, long-term stability, and reduced power consumption. The molecules attached to the surface of the nanowires induce changes in their characteristics because of the nanoscale diameter and high surface-volume ratio [123]. Numerous cutting-edge sensor designs have been established for the production of nanowire sensors and platform integration in order to address the rising need for multifunctional molecular detection and monitoring. For instance, hydrogen and biomolecules have been detected using chemical resistance sensors coupled with metal nanowire networks (e.g., enzymes, glucose, etc.) [124,125]. Single nanowire sensors for the detection of gaseous molecules and charged compounds have been developed using semiconductor nanowires (e.g., metal cations, DNA, RNA, viruses, etc.) [126,127,128].

Lv et al. proposed a dual-channel PCF multifunctional biosensor based on LSPR. The sensor characteristics were modeled and determined by the finite element method [23]. To monitor the analyte’s temperature and RI, a flat two-channel surface was covered with a gold layer, and two nanowires were positioned on the fan-shaped aperture. With its high sensitivity and resolution, the multifunctional PCF-SPR biosensor has great potential for bioanalysis and medical monitoring. By affixing the Au nanowires onto the optical fiber and filling the Au nanowires with silicon, the coupling amount was increased dramatically. The silicon coating enhanced coupling by increasing the depth of penetration, thereby increasing sensitivity. Sensitivity also increases with increasing silicon thickness, but this behavior is limited by the expansion of the SPR spectrum [129]. This type of biosensor can be used as a potential candidate for organic analyte detection in the medical field.

**Figure 6:**
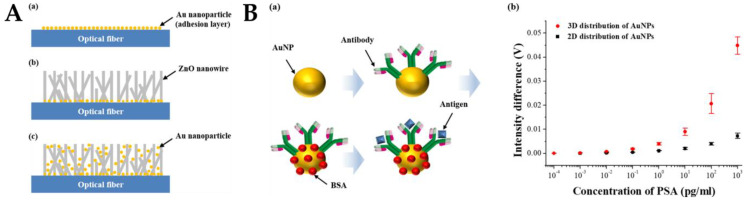
(**A**) Schematic diagram of ZnO nanowire and AuNPs modified sensing probes. In 2D and 3D structures (**B**)(a) diagram of antibody—antigen interaction, and (**B**)(b) diagram of each sensor’s detection results according to various PSA concentrations. Reprinted with permission from Scientific Reports, Copyright 2019, Springer Nature [8].

Kim et al. developed a 3D nanostructured LSPR-based biosensor for prostate-specific antigen (PSA) monitoring [8]. The researchers innovatively used optical fibers instead of the usual silicon wafers and glass substrates [130,131]. ZnO nanowires were synthesized on the fiber surface as supporting materials, and AuNPs were immobilized on the nanowires. Figure 6A depicts the overall fabrication process of the FO-LSPR sensor with 3D nanoparticle arrays and ZnO nanowire supports. Figure 6A(a) shows the first step of forming an adhesive layer composed of AuNPs for adsorbing ZnO nanoparticles as a seed layer on the fiber. The fabricated structure in this step was then used as a 2D FO-LSPR sensor for comparison with the 3D sensor. Figure 6A(b) shows that 200 nm ZnO nanowires were grown by hydrothermal synthesis. In this range, the substrate effect was reduced, and the plasma effect was not significantly reduced. Figure 6A(c) depicts AuNPs fixed on a 3D carrier made of ZnO nanowires. With the weakening of the substrate effect and the increase in the influence of the electric field strength, the efficiency of the LSPR can be improved by stripping the nanoparticles from the fiber surface through the nanowires. Figure 6B(a) is a schematic representation of antibody-antigen interactions and measurement results of the 2D and 3D structures. The 2D and 3D FO-LSPR sensors were used to measure the differences in intensity before and after antibody-antigen reaction at different levels of PSA. In Figure 6B(b), the measurable concentration ranges of the 2D and 3D FO-LSPR sensors were 0.01 pg/mL–1 ng/mL and 0.001 pg/mL–1 ng/mL, respectively. The measured value was 0.001 pg/mL when using the 2D sensor and 0.0001 pg/mL when using the 3D sensor. Due to the relatively large strength of LSPR, the measurement range of the 3D structure was enhanced. The LoD of the 2D and 3D FO-LSPR sensors were 2.06 and 0.51 pg/mL, respectively. Compared with the 2D distribution of nanoparticles, the LoD of the 3D distribution of nanoparticles increased by 404%, and the sensitivity increased by about 171%.

### 3.2. Nanotubes

Since the discovery of carbon nanotubes (CNTs), researchers have studied these 1D materials extensively. CNTs can be thought of as a derivative of graphene, separated by hundreds of nano-spacers to form dense vertically oriented graphite arrays. CNTs have a high surface area due to the particularity of the nanostructure. The high specific surface area provides many binding sites for Au or other nanoparticles to achieve a uniform and dense distribution across the substrate surface. In addition, CNTs have good biocompatibility, which is one of the most important criteria for the development of biosensor probes [132,133]. CNTs are commonly regarded as ideal building blocks for biosensors due to their high width-to-surface-area ratio, large surface area, remarkable thermal and chemical stability, strong mechanical strength, and superb optical and electrical capabilities. CNTs are at the cutting edge of biosensor technology because of their exceptional sensitivity, signal-to-noise ratio, minimal background, wide absorption spectrum, unlabeled detection, and real-time tracking abilities. Using them as biomolecular “stationary bases” may enhance signal transduction and subsequent identification. CNTs may function as nanoscale field transistors due to their semiconductor characteristics [134,135,136]. They are also great alternatives for optical biosensors because of their superb luminescence characteristics and high luminescence intensity. Based on their atomic structure, CNTs can be classified as either single-walled (SWCNTs) or multi-walled (MWCNTs). The large specific surface area of CNTs provides many reaction sites that can interact with a large number of biomolecules [137,138].

**Figure 7:**
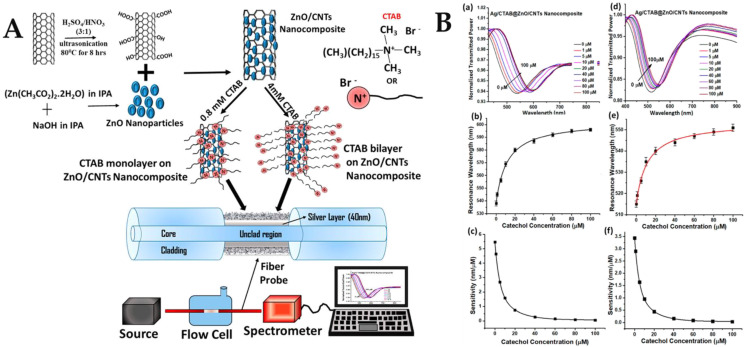
(**A**) Schematic diagram of synthesis of functionalized CNTs and ZnO nanoparticles, attachment of ZnO nanoparticles to the CNT wall, CTAB functionalization of ZnO/CNT nanocomposites with two concentrations, and the fiber probe and experimental setup. (**B**)(a) SPR curve; (**B**)(b) calibration curve; and (**B**)(c) sensor sensitivity for sample pH = 9.5 and CTAB concentration = 4 mM. (**B**)(d) SPR curve; (**B**)(e) calibration curve; and (**B**)(f) sensor sensitivity for sample pH = 7.0 and CTAB concentration = 0.8 mM. Reprinted with permission from ACS Applied Nano Materials [140], Copyright 2020, American Chemical Society.

In the multilayer sensing system, CNTs were deposited over fiber to analyze the RI sensing characteristics. CNT coatings can effectively improve the electric field intensity of the film. This makes CNT-based SPR sensors more sensitive than traditional optical fiber SPR sensors based on metal films [139]. CNT-based SPR sensors have the potential to revolutionize the sensor market by enabling the development of more sensitive, dependable, lightweight, and compact sensors. Researchers may use an SPR sensor based on CNTs to measure the thickness of the film and analyte impurities in accordance with the demands of the present situation. The CNT-based sensor is more reliable than the SPR-based sensor, which is thought to be extremely sensitive. In 2020, Pathak proposed the application of CNTs for the development of fiber optic biosensors and proposed a fiber optic plasma sensor based on Ag film/ZnO/CNTs for the detection of catechol [140]. The interaction of catechol with ZnO/CNTs nanocomposites plated on a silver film of the sensing probe resulted in the variation of SPR resonance parameters. The schematic diagram of the portable experimental setup and the synthesis and functionalization of the ZnO-modified CNT nanocomposites are shown in Figure 7A. The thickness of the sensing layer on the metal-coated region was directly related to the sensitivity of the sensor because it controlled the interaction of the evanescent field with the analyte. Theoretically, the highest sensitivity can be obtained for the sensing layer thickness that provides the maximum electric field strength at the sensing layer-analyte sample interface. This is depicted as the maximum shift in the resonance wavelength of the SPR spectrum due to a fixed change in analyte concentration for a given thickness. As the catechol concentration increased, more and more molecules were attracted to the probe and were oxidized due to the catalytic activity of the ZnO/CNT nanocomposite. This altered the physicochemical properties of the silver layer and thus the effective RI around it. The resonance wavelength was shifted to higher values as more catechol molecules were adsorbed onto the nanocomposite layer when the pH was 9.5 and the CTAB was 4 mM. For a concentration change from 0—100 μM, a total offset of 58 nm was achieved, as shown in the SPR curve in Figure 7B(a). A calibration curve was plotted between the resonance wavelength and catechol concentration in Figure 7B(b). Figure 7B(c) shows that the highest sensitivity of 5.46 nm/μM was obtained at the lowest concentration of catechol. The SPR curve when the pH was 7.0 and the CTAB was 0.8 mM is shown in Figure 7B(d), the calibration curve is shown in Figure 7B(e), and the sensitivity curve of the 7B(f) sensor shows that the sensitivity was 3.44 nm/μM. The LoD of the probe was 0.9 μM and 0.1 μM in two cases, respectively. These LoD are satisfactory in the range suitable for practical applications, and in addition to being stable and durable, the current method has higher sensitivity compared to enzyme probes.

So far, nanotubes have not been widely used in the development and application of fiber optic biosensors. With their high biocompatibility, nanotubes have been widely and effectively applied in the field of electrochemical sensing and SERS sensing. However, the application of nanotubes in the field of fiber optic biosensors has not attracted the attention of researchers. It is believed that CNTs will be commonly applied in the field of fiber optic biosensors in the future.

### 3.3. Other 1D Nanomaterials

Currently, nanorods are also widely used in the development of optical biosensors. Fallah et al. reported a ZnO nanorod-based fiber optic biosensor for the rapid and sensitive detection of Escherichia coli (*E. coli)* [59]. After applying a thin coating of AuNPs (about 50 nm) to the tip of the multi-mode plastic fiber, ZnO nanorods were grown on the Au layer. Within the first 10 s of interaction between contaminated water and *E. coli*, the sensor displayed a very quick reaction. Researchers investigated *E. coli* concentrations ranging from 1000–4000 CFU/mL. There was a clear pattern in the sensitivity. The sensing platform has tremendous potential for routine monitoring of the presence of different harmful bacteria in water and food.

Mao et al. developed a novel fiber laser biosensor based on Au nanorods and SiO_2_ nanoparticle functionalized thin-walled hollow fibers for the sensitive detection of horseradish peroxidase (HRP) [51]. The high sensitivity was due to the high surface area-to-volume ratio of the nanomaterials. The streptavidin-biotin coupling mechanism acts as a bridge for enzyme-specific recognition. Compared to the control group without nanomaterials, the nanoparticle functionalized fiber flow control lasers had a lower laser threshold. The experimental results showed that the nanomaterials, including gold nanorods (AuNRs) and SiO_2_NPs, helped increase the binding sites of the optical fluidic laser and reduce the laser threshold. Enzyme assays have been shown to have higher sensitivity for both nanomaterials due to the high surface-to-volume ratio. Sensitive HRP detection was achieved in the range of 75–15,000 pM with good linearity. The results showed that the sensitivity of this method increased by 57.4%. The AuNRs of the SiO_2_NPs-based optical fiber fluidic laser biosensor had little improvement due to its absorption effect. Therefore, it is promising to develop high-performance biological detection technology based on optical fiber flow-controlled lasers using nanomaterial strategies.

## 4. Application of 2D Nanomaterials in the Field of Optical Fiber Biosensors

Compared with traditional biosensors, the electron transfer efficiency, surface area, and biocompatibility of sensors made of 2D materials are much higher as the materials enhance the performance of optical fiber biosensors. Optical signals produce EWs that enter the optically thinner medium when it completely reflects off the optical fiber and metal film surfaces. This optically thinner medium contains a specific plasma wave. The collision of the two waves causes resonance. The energy of the reflected light significantly diminishes when the EWs resonate with the surface plasma wave because the surface plasma wave absorbs the majority of the incoming light’s energy. Therefore, metal films are usually used to excite SPR for the detection of biological substances. Compared with the SPR of traditional metal films, the SPR of 2D nanomaterials is very sensitive to the change in the optical phase. Based on this principle, some biological probes are modified on the surface of 2D materials. When they capture the target molecules, the phase change of SPR at the interface of the 2D materials is very drastic, which can realize the highly sensitive sensing of a series of biological molecules. Optical biosensors have more stable signals and can avoid the interference of temperature and other factors. Therefore, some typical 2D nanomaterials such as GO, MXene, and MoS_2_ are favored by researchers. All-fiber photonics, photo-electronics, and optical biosensors have been successfully combined in recent years, thanks to the widespread usage of graphene and other 2D materials. The use of 2D materials, which can be used to control the polarization, phase, intensity, and frequency of light beams as well as to realize active photoelectric conversion and electro-optic modulation, improves the interaction between light and matter in silica fiber devices. This opens new possibilities for the study and development of optical fiber biosensors. This section primarily examines the fiber optic biosensor applications of a few well-known 2D materials.

### 4.1. Metal Films

In recent years, increasingly sensitive and unlabeled optical device-based fiber SPR using metal thin films and nanostructures have been developed [141]. SPR is an optical phenomenon generated by the propagation and collection of free electron oscillations in metal films and nanostructures surrounded by dielectric media [27]. EWs form and enter into the photophobic medium, and there is a certain plasma wave in the medium when the total reflection of light occurs on the surface of the optical fiber and metal film. Resonance may occur when the two waves meet. The detected reflected light intensity is greatly weakened when EWs resonate with surface plasmon waves. The energy is transferred from the photon to the surface plasma, and most of the energy of the incident light is absorbed by the surface plasma wave, which drastically reduces the energy of the reflected light. With a variety of fiber architectures, biosensor probes for depositing metal films and NPs have been developed (e.g., side-polished, tapered, U-shaped, hyper-fiber, nanofiber, etc.) to generate strong plasma-matter interactions. SPR sensors are sensor probes that generate plasma using metal films and nanostructures and are applied to light fields to detect chemical, physical [142], biomolecular [143], DNA [41], and microbial agents. In order to improve the sensitivity of the sensor, several optical geometries have been studied and developed. Recent sensor fabrication technologies indicate that the combination of nano-deposition, thin film coating, and optical fiber is a hot topic for measuring RI.

**Figure 8:**
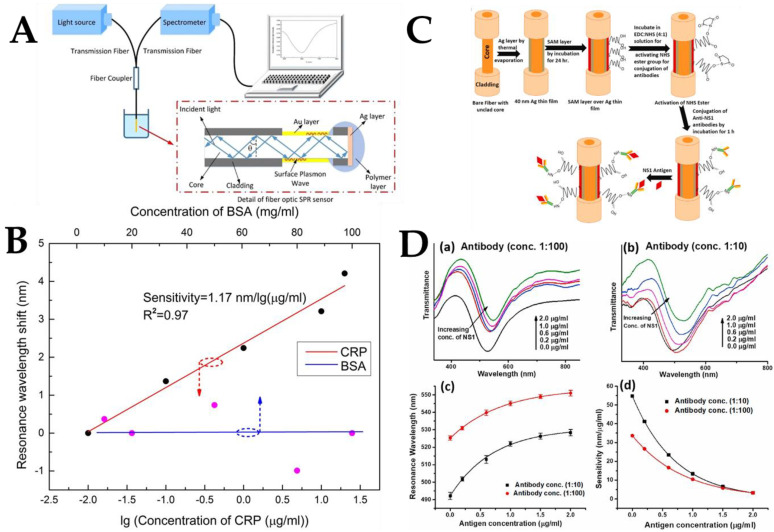
(**A**) Schematic diagram of experimental device. (**B**) Resonance wavelength shifts are plotted as logarithmic functions of CRP and BSA concentrations. Reprinted with permission from Scientific Reports, Copyright 2017, Springer Nature [144]. (**C**) Fabrication steps of the fiber optic SPR probe. SPR spectra were used for antibody concentrations (**D**)(a) 1:100 and (**D**)(b) 1:10 as the NS1 antigen concentration increased. Corresponding (**D**)(c) calibration curve and (**D**)(d) sensitivity curve. Reprinted with permission from Biosensors and Bioelectronics, Copyright 2022, Elsevier [145].

Wang et al. proposed and demonstrated an unlabeled fiber optic SPR biosensor for the specific detection of C-reactive protein (CRP) [144]. The uncoated MMF was used as the sensing area for the fiber optic sensor, which was then fabricated by depositing an Au film. The sensitivity of the developed sensor was then assessed by detecting various concentrations of NaCl solutions. Figure 8A depicts the SPR probe and measurement equipment in detail. The anti-CPR monoclonal antibody was fixed on the surface of the sensor to provide CPR-specific tests with the use of the biological crosslinked membrane (polydopamine). Further, the anti-CRP monoclonal antibody was subsequently linked to the sensor surface for CPR-specific detection. The experimental results, as shown in Figure 8B, were obtained by optimizing the fixation time of the anti-CRP monoclonal antibody and antigen-antibody reaction time. The results showed that the sensor displayed a reasonable linear (R^2^ = 0.97) response in the CRP concentration range of 0.01–20 μg/mL. The maximum CRP sensitivity under the circumstances was 1.17 nm/lg(μg/mL). While the resonance wavelength shifted in BSA detection, it increased with the increase in CRP concentration in the concentration range of 10–100 mg/mL. The anti-CRP monoclonal antibody immobilized sensors did not show non-specific detection, as evidenced by BSA detection. According to the experimental findings, when CRP concentration increased, the resonance wavelength shift also increased.

Gahlaut et al. coated the fiber with a silver film and dengue biomarker NS1 protein antigen and developed a fiber optic SPR biosensor for dengue virus detection by studying various self-assembled monolayers (SAM) with different chain lengths and surface parts to optimize the surface motifs attached to anti-NS1 antibodies. Figure 8C depicts the structure of the fiber, the steps of probe fabrication, and the mechanism of interaction with the antigen. This type of sensing probe was used for the quantification of the NS1 antigen. NS1 antigen detection depends on the interaction of the NS1 antigen present in the infected serum sample with the anti-NS1 antibody attached to the fiber probe with the help of the SAM layer on the silver film. When the NS1 antigen in the sample solution binds to the antibody on the probe, the RI around the silver layer changes, which is manifested as a change in the resonance wavelength of the SPR spectrum. During probe fabrication, the antibody concentrations were first changed to 1:100, 1:50, 1:10, and 1:5. The results showed that the probe with an antibody concentration of 1:10 showed the best performance in terms of resonance wavelength shift in the above NS1 antigen concentration range. The reasons for this trend can be understood as follows: lower antibody concentrations may not be sufficient to have sufficient sensing sites on the probe; however, high concentrations may cause steric hindrance between adjacent antibodies, resulting in reduced sensing performance. Figure 8D shows the detection response at two antibody concentrations of 1:100 and 1:10. For both probes, a redshift in resonance wavelength was observed as the antigen concentration increased from 0–2 μg/mL, as shown in Figure 8D(a,b). The redshift of the resonance wavelength means that the RI around the Ag layer increased when the NS1 antigen was bound to its antibody on the probe surface. Calibration curves describing the resonance wavelength as a function of NS1 antigen concentration are shown in Figure 8D(c) for the 1:100 and 1:10 probes. The experiment was repeated five times for each probe to find the average resonance wavelength, which is shown as the standard deviation of the error line in the calibration plot. It can be noticed from Figure 8D(d) that when the antibody concentration increases, the resonance wavelength will be blueshifted. In SPR-based sensors, the resonance wavelength shift is related to the change in RI around the metal layer. The probe gives a very satisfactory response to real samples and can work in a physiological range with high sensitivity and selectivity, demonstrating the feasibility of its clinical use [145]. In the field of food safety detection, optical fiber SPR sensors coated with Ag films are also used for the detection of glucose and fructose. A simple, rapid, unlabeled technique can be used to detect glucose/fructose in pure honey.

Hossain et al. used the finite element method for the numerical and theoretical analysis of a hollow PCF-SPR biosensor. The sensor based on plasma metal and an Ag layer has high sensitivity and low constraint loss and can achieve a long-time detection of 1.33–1.42 RIU. Therefore, SPR sensors can be used for the detection and sensing applications of unknown analytes, such as biomolecular components, lipids, proteins, and carbohydrates [24]. In another study, Wang et al. used an Au film-modified fiber U-shaped structure cascaded multi-channel fiber SPR sensor to detect glucose and sucrose [15]. The sensor is useful for the simultaneous measurement of numerous parameters and may be immediately put into a tiny space for measurement. Further, Zhang et al. also proposed a glucose detection platform based on the MMF-SMF-MMF structure of SPR and enzymatic reaction, providing a new way for the detection of food additives and medical immunoassays [5]. The dual-parameter SPR biosensor was developed by Zheng et al. based on the HPCF modified with an Au film and AuNPs. The developed sensor offers a fresh approach to dual-parameter measurement and broadens the scope of applications for optical fiber biosensors in the biomedical industry [146]. However, the low detection range reduces the practical value of dual-parameter sensors to a certain extent. A double-layer Cu fiber optic biosensor based on SPR has been proposed and verified experimentally [147]. On a polished optical fiber surface, a uniform copper layer was deposited using an electron beam-assisted sputtering technique. The thickness of the deposited Cu film was found to be 50.9 nm and 51.3 nm, respectively. Due to the high RI and unique photoelectric characteristics of Cu, the biosensor based on Cu film SPR has been applied to detect various concentrations of bovine serum albumin (BSA) solution and has achieved satisfactory sensitivity (1.907 nm/(mg/mL)) and LoD (5.70 × 10^−7^ mg/mL). Notably, this biosensor shows sensitivity to sub-microliter doses, promising biochemical applications in DNA hybridization, cancer screening, medical examination, and environmental engineering.

Metal thin films of Au, Ag, and Cu are widely used in the development of biosensor probes based on fiber optic SPR. Some recent research results of SPR biosensors based on metal film modification are shown in Table 3. The application of metal composite film in the field of fiber optic biosensors can be considered in the future development of sensor probes.

### 4.2. Graphene Oxide

Graphene-based nanomaterials have significant and independent optical properties, including tunability and broadband adsorption, as well as influence dependent on material polarization [149,150]. The development of optical system-based biosensors has been facilitated by the distinctive optical characteristics of materials based on graphene.

**Figure 9:**
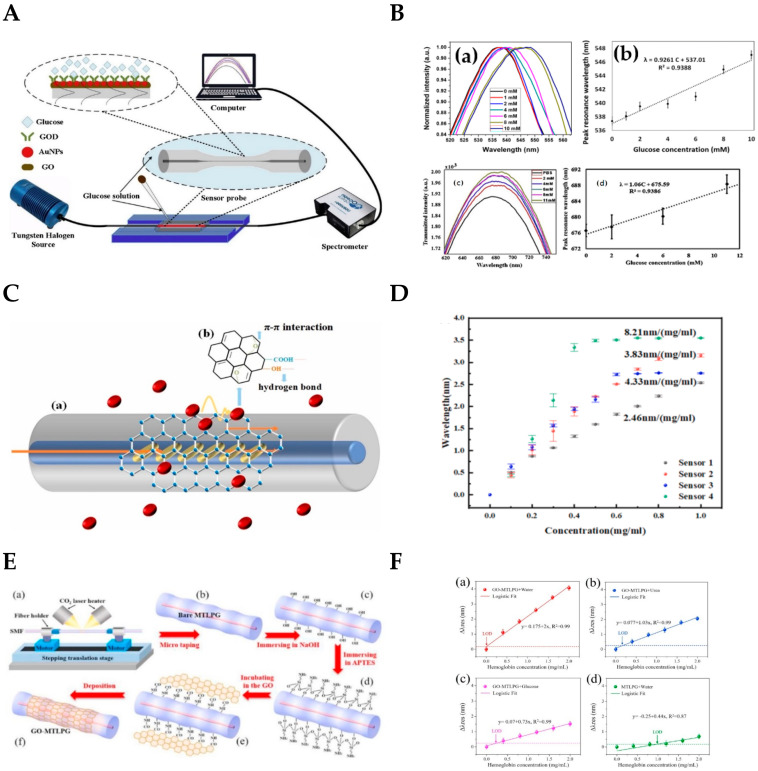
(**A**) Schematic diagram of LSPR fiber biosensor based on GO coating. Reprinted with permission from Optik, Copyright 2020, Elsevier [152]. Unmodified LSPR sensor (**B**)(a) transmission spectrum and (**B**)(b) linear graph of the sensor. Modified LSPR sensor (**B**)(c) transmission spectrum and (**B**)(d) linear graph of the sensor. Reprinted with permission from Plasmonics, Copyright 2019, Springer [57]. (**C**) Schematic diagram of fiber optic biosensor based on GO-coated Ex-TFG. (**D**) Sensitivity of biosensors with different GO levels. Reprinted with permission from Sensors and Actuators B: Chemical, Copyright 2022, Elsevier [28]. (**E**) Schematic diagram of the surface of the fiber sensing probe decorated with GO nanosheets. Hb in (**F**)(a) DI water; (**F**)(b) Urea; and (**F**)(c) glucose solution with GO-MTLPG. Hb in (**F**)(d) DI water with MTLPG. Reprinted with permission from Optical Materials, Copyright 2020, Elsevier [153].

In 2018, Wang et al. verified the effect of GO on improving sensitivity in the development of an SPR immunosensor. The results show that the sensitivity of the GO-modified fiber probe was 68% higher than that of the unmodified fiber probe [151]. In 2019, Yang et al. proposed a glucose biosensor probe based on LSPR for glucose detection at a concentration of 0–10 mM and achieved a sensitivity of 0.93 nm/mM [57]. Later, Yang et al. used GO to modify the previous LSPR-based sensor probe in order to improve the sensitivity of the biosensor [152]. The fiber probe was tested by the device shown in Figure 9A. The comparison of the two results is shown in Figure 9B. Figure 9B(a,b) are the results of the LSPR sensor probe without GO modification, and Figure 9B(c,d) are the results of the LSPR sensor probe with GO modification. As can be seen from the Figure 9B, the detection linear range of the sensor probe modified by GO changed from 0–11mm, and the sensitivity increased from 0.93 nm/mM to 1.06 nm/mM. The slight increase in sensitivity demonstrates the feasibility of GO in the development of LSPR-based biosensors.

The layers of GO also have an impact on the sensitivity and detection range of biosensor probes. Sun et al. investigated a hemoglobin (Hb) biosensor using an excessively tilted fiber grating (Ex-TFG) coated with GO [28]. The sensing system included a biosensor based on Ex-TFG coated with GO monolayers for the detection of Hb biomolecules, as shown in Figure 9C. The GO layer on the Ex-TFG had a high surface-to-volume area and abundant functional groups, which was conducive to the adsorption of biomolecules. Fiber optic sensing relies on changes in the evanescent field to measure RI changes. The Hb biomolecules adsorbed by the GO layer through π-π interaction and hydrogen bond interaction can greatly induce the perturbation of the grating evanescent field, leading to the detection of the wavelength shift of the cladding mode resonance of Ex-TFG. GO was adsorbed onto the surface of the Ex-TFG sensor probe through π-π and H-bond interaction. Then the sensitivity of the Hb biosensor with different layers of GO coating was tested. The results indicated that GO coating might increase the Ex-TFG’s bioactivity and render Ex-TFG responsiveness to the Hb biomolecular solution. Figure 9D shows that three biosensor probes had sensitivity values of 3.83 nm/(mg/mL), 4.33 nm/(mg/mL), and 8.21 nm/(mg/mL), respectively. Unfortunately, the detection range was drastically decreased and changed to 0.8 mg/mL, 0.6 mg/mL, and 0.4 mg/mL, respectively.

Wang et al. demonstrated a label-free biosensor platform using GO nanosheet functionalized micro-taper long period grating (MTLPG) for hemoglobin detection in various solvents [153]. Figure 9E shows the schematic diagram of the GO nanosheets decorated on the surface of the fiber sensing probe. As the hemoglobin molecule is absorbed onto the surface of the fiber, the resonance wavelength shifts, allowing detection of the hemoglobin concentration. As shown in Figure 9F(a–c), the sensitivities reached −2 nm/(mg/mL), −1.03 nm/(mg/mL) and −0.73 nm/(mg/mL) in the range of 0–2.0 mg/mL for hemoglobin concentration in DI water, urea solution, and glucose solution, respectively. Figure 9F(d) shows a control group without GO-modified probes. It can be concluded that the inherent excellent optical and biochemical properties of GO can provide high stability, strong light wave interference, and excellent biocompatibility for the biosphere surface layer.

Currently, GO and other fiber optic biosensor probes have been developed for glucose and hemoglobin sensing. In the future, GO will have a broad prospect in the field of fiber optic biosensors. Table 4 summarizes some research results of fiber optic biosensor probes modified by GO.

### 4.3. Molybdenum Disulfide

Molybdenum is one of the trace dietary elements necessary for human survival. Aldehyde oxidase, sulfite oxidase, and xanthine oxidase are MO-containing enzymes involved in key metabolic activities of the human body. Therefore, some molybdenum-based compounds have been widely used in biomedical research because of their good biocompatibility [159,160,161]. MoS_2_ consists of S-Mo-S bonded by weak van der Waals forces and is known as a 2D nanocrystalline material “beyond graphene” [162]. MoS_2_ has several advantages compared to graphene, including greater efficiency at optical absorption, a wider band gap, greater electron mobility, higher surface-to-volume ratio, less poisonous, and biocompatible. As a result, it has been successfully used for sensing purposes. MoS_2_ nanomaterials have attracted more and more attention in biomolecular detection [7,163], bacterial analysis [19,103,164,165], virus detection [166,167], and cancer diagnosis [160,168,169,170,171] due to their unique physical and chemical properties. Due to easy modification and large specific surface area, MoS_2_ can adsorb a variety of biomolecules and drug molecules through covalent or non-covalent interactions, increasing the stability of its protein targets as well as the accuracy and sensitivity of detecting particular biomarkers [161,172,173].

Kaushik et al. developed an antibody-functionalized fiber optic SPR sensing probe for the unlabeled detection of BSA [174]. Two kinds of BSA sensor probes were prepared with MoS_2_ and without MoS_2_ modification. The experimental results showed that compared with the fiber SPR sensor without MoS_2_, the LoD of the MoS_2_-modified sensor showed a significant decrease from 0.45 μg/mL to 0.29 μg/mL. Thus, MoS_2_ can be used to reduce LOD and improve sensitivity for the development of fiber sensing probes.

**Figure 10:**
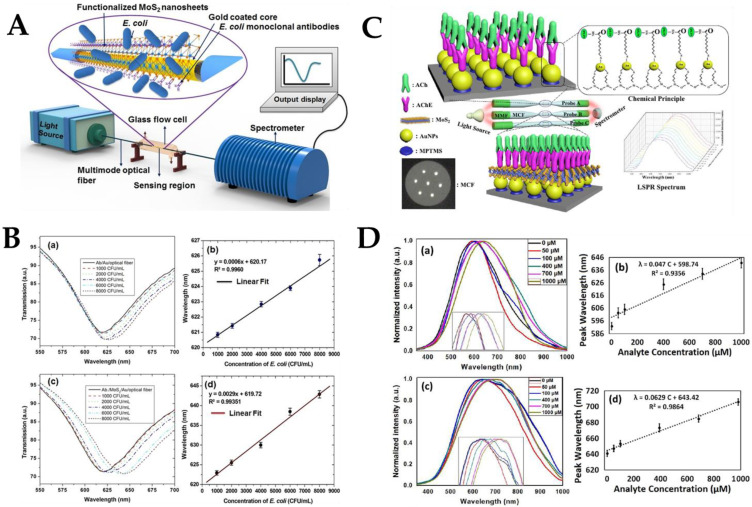
(**A**) Schematic representation of the experimental setup of the developed fiber optic SPR immunosensor. (**B**)(a) SPR spectrum of Ab/Au immunosensor; (**B**)(b) resonance wavelength of Ab/Au immune-sensor; (**B**)(c) SPR spectra of Ab/MoS_2_/Au immunosensor; and (**B**)(d) resonance wavelength of Ab/MoS_2_/Au immunosensor. Reprinted with permission from Biosensors and Bioelectronics, Copyright 2019, Elsevier [165]. Probe 1: (**D**)(a) LSPR spectrum with AuNPs and acetylcholinesterase. (**C**) Schematic of the multicore optical fiber-based LSPR detection system. (**D**)(b) linearity plot. Probe 2: (**D**)(c) LSPR spectrum with AuNPs, MoS_2_ and acetylcholinesterase, (**D**)(d) linearity plot. Reprinted with permission from IEEE Transactions on Instrumentation and Measurement, Copyright 2021, IEEE [163].

Since then, some researchers have used MoS_2_ for bacteria or virus detection. MoS_2_ nanosheets were fixed to the Au membrane interface of an SPR immunosensor via Au-S bonding and then bio-conjugated effectively with *E. coli* monoclonal antibody through hydrophobic interaction [165]. *E. coli* with a population density of 1000–8000 CFU/mL was detected without label. The results showed that the proposed SPR immunosensor platform could sensitively detect *E. coli* as low as 94 CFU/mL. The target analyte (*E. coli*) has been developed for the immunosensor to achieve accurate and selective detection in the presence of other interfering bacteria. However, the developed fiber optic SPR system has some limitations, including high cost, short effective time, and other shortcomings. In order to study the spectral characteristics of the fiber optic SPR sensor, the resonance wavelength obtained after the antibody was fixed onto the surface of the SPR immunosensor was considered as the reference resonance wavelength. Different concentrations of *E. coli* were detected using the SPR sensing device shown in Figure 10A. The SPR spectrum is shown in Figure 10B(a). It can be observed that with the increase of *E. coli* concentration, the resonance intensity in the SPR spectrum decreased and showed a redshift, thus determining the resonance wavelength of each concentration. Figure 10B(c) shows the corresponding change of Ab/MoS_2_ at the resonance wavelength and *E. coli* concentration. The resonance wavelength rose linearly with *E. coli* concentration in the range of 1000–8000 CFU/mL for the Ab/Au/fiber and Ab/MoS_2_ immunosensors, as can be observed from Figure 10B(b,d). When *E. coli* and monoclonal antibodies interact on the Au layer of the MoS_2_ functional SPR immunosensor, the RI of the environment changes due to the formation of an antibody-antigen complex, which also changes the EWs characteristics of the interaction. The amount of *E. coli* in the buffer solution was directly related to the change in RI. The Ab/MoS_2_/Au fiber exhibited a greater wavelength shift, improving sensitivity of the sensor. The functionalized nanosheet’s huge surface area boosted the antibody’s binding density, which resulted in more target analytes being captured.

MoS_2_ not only plays a role in improving sensitivity and reducing detection limits in the bacterial sensor probe but also plays an important role in other fiber optic biosensors. Zhu et al. proposed a biosensor probe based on LSPR for the detection of acetylcholine [163]. A controlled experiment was set up to control a single variable to verify that MoS_2_ improved the performance of biosensor probes. As shown in Figure 10C, AuNPs/acetylcholinesterase and AuNPs/MoS_2_/acetylcholinesterase were used to modify fiber sensing probes with different structures, respectively. An AuNPs and acetylcholinesterase functionalized MMF-tapered MCF-MMF was used as sensing probe 1, and an AuNPs, MoS_2,_ and acetylcholinesterase functionalized MMF-tapered MCF-MMF was used as sensing probe 2. The LSPR sensor spectrum and resonance wavelength fitting of probe 1 are shown in Figure 10D(a,b), and the LSPR sensor spectrum and resonance wavelength fitting of probe 2 are shown in Figure 10D(c,d). As shown in Figure 10D(b,d), in comparison to probe 1, probe 2 had a higher linear fit, higher sensitivity (increased by 33.8%), and lower LoD (14.28 μM).

MoS_2_ possesses a unique resilience, electron mobility, and huge surface-to-volume ratio in its lattice structure. It is important to emphasize that adding a spatial structure with a sizable specific area would raise the target biomolecule’s number of interaction sites and boost the probe’s sensitivity. So far, it has been proved that MoS_2_ modification of sensor probe surfaces can effectively improve sensor performance. MoS_2_, as a 2D nanomaterial of transition metal dichalcogenides (TMDCs), has set off a new trend in the field of biosensor research. However, in the field of fiber optic biosensors, there have been no spectacular achievements. In the future, fiber optic biosensor probes will gradually enter the field of clinical research. Table 5 lists some of the achievements of MoS_2_ applied in the field of fiber optic biosensors.

### 4.4. MXene

MXene, as a novel 2D transition metal carbide and nitride nanomaterial, is characterized by its unique morphology and properties, such as good electrical conductivity, high surface area, excellent hydrophilicity, and abundant surface functional groups [175,176,177,178,179]. The general formula for MXene can be expressed as M_n+1_X_n_Tx (n = 1–4), where “M” is a transition metal, “X” is C or N, and “T” is a surface terminal, such as -O, -F, or -OH. Compared to graphene, MXene exhibits a large number of hydrophilic functional groups on its surface, which can effectively trap molecules in an aqueous solution. Synthesis of MXenes and exploitation of their inherent properties have been explored in the field of biosensors to achieve high sensitivity and high selectivity. The unique characteristics of MXenes, especially their optical properties and good biocompatibility, make them a good candidate for biosensor construction. In the last five years, MXenes have made remarkable progress in the field of sensing and biosensor analysis devices based on different technologies proposed by researchers. In addition to its applications in photoluminescence and colorimetric sensing, MXenes-based materials have now been explored in SPR, SERS, and LSPR fiber sensing. Recently, a wide range of biomedical applications has been found in biosensors. Previous studies have shown that Ti_3_C_2_MXenes have inherent peroxidase-like activity [180,181]. However, the catalytic performance of MXenes alone still lags behind other nano-enzymes such as metals and metal oxides. Therefore, developing easy-to-use methods to manufacture MXenes nanomaterials with high catalytic efficiency remains a challenge.

**Figure 11:**
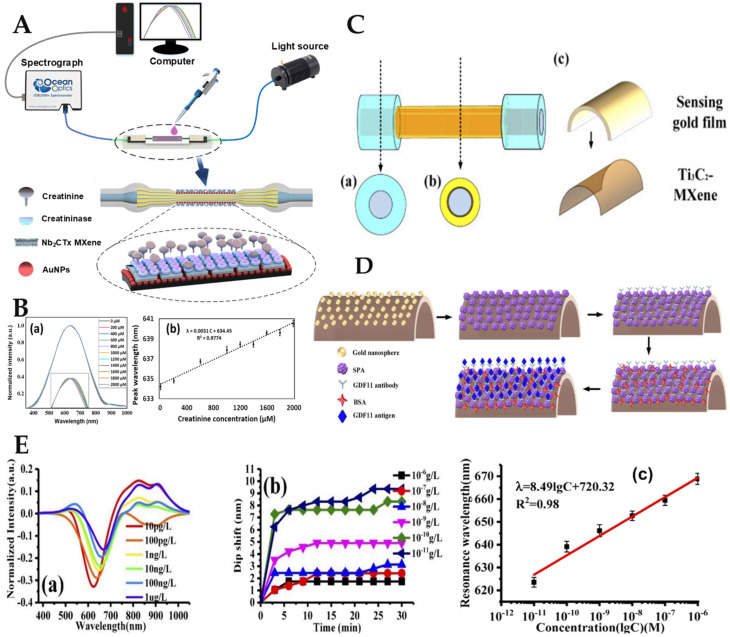
(**A**) Diagram of CTC structure sensing probe and measuring device. (**B**)(a) LSPR sensing spectrum and (**B**)(b) proposed sensor linearity plot. Reprinted with permission from Optics Express, Copyright 2022, Optica [16]. (**C**) Optical fiber structure diagram. (**D**) Structure diagram of the sensing probe. (**E**)(a) SPR spectra; (**E**)(b) resonance wavelength shift with time; and (**E**)(c) relationship between the resonance wavelength and the logarithm of the solution concentration. Reprinted with permission from Optics Express, Copyright 2021, Optica [176].

It is worth mentioning that 2D niobium carbide (Nb_2_C) MXene nanomaterials are highly biocompatible, biodegradable, and have an inherent light response. Therefore, Nb_2_CTx MXene is also widely used in the development of biosensor probes [16,178,182]. Li et al. decorated the convex fiber-tapered MCF-convex fiber (CTC) structure with AuNPs and Nb_2_CTx MXene and developed a biosensor based on the LSPR effect for the detection of creatinine substances [16]. The creatinine biosensing probe model diagram and its detection schematic diagram are shown in Figure 11A. The strong EWs were obtained by tapering the total internal reflection transmission mode of the fiber probe to induce the LSPR effect of AuNPs, so as to improve the probe sensitivity. Nb_2_CTx MXene adsorbed more creatinase enzyme to enhance the specific recognition ability of the probe. Creatinine was adsorbed on the surface of the sensing probe to undergo biochemical decomposition, resulting in RI changes in the surrounding medium, and the resonance wavelength gradually redshifted. The results showed that the wavelength shift increased with increasing creatinine concentration. The LSPR sensor spectrum is shown in Figure 11B(a) by testing sample solutions with different concentrations of creatinine. After analyzing the above detection data, the relationship between sample solution concentration and resonance wavelength is shown in Figure 11B(b). The sensitivity of the biosensors based on LSPR was 3.1 pm/μM, and the LoD was found to be 86.12 μM, respectively.

MXene not only plays a positive role in the field of human health detection but also attracts researchers’ attention in the field of water quality monitoring. Kumar et al. proposed a highly sensitive SPR sensor based on D-shaped PCF (DPCF) of Ti_3_C_2_Tx MXene with different thicknesses for biomolecular detection [12]. To promote the SPR effect, an MXene/Au mixed metal layer was applied to the DPCFs flat top surface. Simulated biomolecules have RIs between 1.33 and 1.39. According to the findings, in the absence of MXene, the wavelength sensitivity was 2000 nm/RIU. However, the wavelength sensitivity was 7000 nm/RIU and 13,000 nm/RIU, respectively, when the MXene layer was 14 nm and 27 nm thick. According to numerical findings, the MXene films’ wavelength sensitivity rose by 3.5 and 6.5 times, respectively. As a result, the biosensor based on Ti_3_C_2_Tx/Au layered DPCF had a high sensing capability and may be utilized to detect biological and chemical samples with RI values between 1.33 and 1.39. Liu et al. successfully developed an MXene-based biosensor for the detection of biological substances such as GDF11. The proposed fiber SPR sensor was decorated with 2D material Ti_3_C_2_ MXene [176]. The structure of the fiber sensing probe is shown in Figure 11C. Ti_3_C_2_Tx MXene/Au film/AuNPs were used to decorate the plastic-coated multimode fiber to obtain the sensing probe, as shown in Figure 11D. The MXene-modified fiber SPR sensor’s sensitivity was raised to 4804.64 nm/RIU. The LoD of the fiber optic SPR sensor was 0.577 pg/L after functionalization of the GDF11 antibody, which was 100 times lower than the single-molecule enzyme-linked immunosorbent assay (ELISA), enabling the sensor to uniquely identify GDF11. Figure 11E depicts the sensing probe test curve for GDF11 based on SPR. Figure 11E(a) shows that the resonance wavelength and the light intensity decreased when the concentration of GDF11 antigen solution increased. As shown in Figure 11E(b), as detection time rose, the resonance wavelength excursion first increased quickly before gradually becoming steady. The resonant wavelength shift increased with decreased GDF11 antigen solution concentration. Ti_3_C_2_-MXene and AuNPs can effectively improve the sensitivity of the sensor probe. After processing the data of the above detection results, the relationship between GDF11 concentration and resonance wavelength was obtained, as shown in Figure 11E(c). It could accurately detect GDF11 with an LoD of 0.577 pg/L when the fiber SPR biosensor was functionalized with the GDF11 antibody.

Synthesis of MXenes and exploitation of their inherent properties have been explored in the field of biosensors to achieve high sensitivity and high selectivity. The unique characteristics of MXenes, especially its optical properties and good biocompatibility, make it a good candidate for biosensor construction. In the last five years, MXenes has made remarkable progress in the field of sensing, and biosensor analysis devices based on different technologies have been proposed by researchers. In addition to its applications in photoluminescence and colorimetric sensing, MXenes-based materials have now been explored in SPR, SERS and LSPR fiber sensing. The applications of MXene in the field of optical fiber biosensing are listed in Table 6.

### 4.5. Other Novel 2D Nanomaterials

Rahman designed the SPR biosensor using a novel 2D material, tin selenide (SnSe) allotrope fiber [185]. Due to the improved optical features of its 2D graphene-like structure, single-layer SnSe has gained a lot of attention lately as a biomolecular recognition element (BRE) in sensor design. For sensing applications, the sensitivity of BRE fiber sensors with three different single allotrope types was examined. The findings demonstrate that the sensitivity of α-SnSe, δ-SnSe, and ε-SnSe sensors, respectively, were 3225 nm/RIU, 3300 nm/RIU, and 3475 nm/RIU. The findings demonstrate that the suggested sensor’s increased sensitivity outperforms the conventional sensor built on a metal film. In order to create exceptionally sensitive fiber SPR biosensors for DNA hybridization, the suggested SnSe allotrope may be a viable substitute for conventional BRE.

Similar to graphene, TMDC has also attracted a lot of attention. TMDC MoSe_2_ was applied to SPR fiber optic biosensor by Liu et al. for the detection of Goat-Anti-Rabbit IgG [186]. Cysteamine hydrochloride with enrichment groups can generate self-assembled membranes to adhere tightly to the surface of MoSe_2_. In this study, the researchers combined the fast response of the fiber optic SPR sensor with the enhanced sensitivity of the MoSe_2_ nanofilm. A suggested and constructed fiber optic SPR biosensor with a MoSe_2_-Au nanostructure offers impressive sensitivity and LoD. Additionally, scientists tested the bio-affinity of the biosensor using BSA as the target molecule with MoSe_2_ deposition cycles ranging from zero to eight. Finally, the immunoassay was performed with goat anti-rabbit IgG and a LoD of 0.33 μg/mL was reached. The fast response and high bio-affinity indicate that the proposed MoSe_2_-Au SPR immunosensor has strong applicability in specific interactions and immunotherapy.

Tungsten disulfide (WS_2_), a TMDC, also exhibits many unique photoelectric properties, such as a high compound RI ratio, direct band gap, and large surface-to-volume ratio [187]. However, there are relatively few experimental studies on WS_2_ in SPR sensors, which mainly focus on prism-based SPR sensors [188]. WS_2_ modified sensors can improve the sensitivity of fiber SPR sensors. Cai et al. proposed a theoretical model for glucose detection based on an SPR fiber optic biosensor coated with Au/ZnO/WS_2_ multilayer film. Compared with traditional SPR sensors [189], the sensitivity of biosensors may be increased when using WS_2_ materials. In order to load glucose oxidase, WS_2_’s absorptive capability is exploited to create glucose-sensitive areas. Once the solution’s RI has been determined, the glucose concentration may be computed using the connection between the RI and the glucose concentration. The suggested WS_2_-based SPR fiber biosensor has a special function in the measurement of glucose levels. It is clear that WS_2_ modification-based sensors may provide a platform for biochemical detection that is sensitive, inexpensive, straightforward, and environmentally protective.

## 5. Application of 3D Nanomaterials in the Field of Optical Fiber Biosensors

The main advantage of nanoarrays for sensing applications over conventional planar metal films is the strong local field enhancement on the surface of the structure [190]. Therefore, the influence of RI changes in the confined area around nanomaterials on sensing performance is of great significance for biomolecular detection. In order to significantly reduce reflection in the plasmonic mode’s resonant coupling spectrum and to be sensitive to the environment around the probe surface, a 3D nanopore array of the plasmonic sensing element has been inserted into the optical fiber surface. The 3D porous structure has abundant interconnected pores and multiple channels for analyte diffusion, which can effectively promote the uniform and dense adsorption of biological substances by the plasma nanostructure, so as to effectively identify the object to be tested and significantly improve the sensing sensitivity. However, the poor stability of 3D porous structures may easily lead to the collapse or closure of interconnected micropores [58,191].

By adjusting the chemical potential of the graphene, the researchers were able to detect several SPR absorption bands on graphene bands for biosensors composed of graphene ribbons [192]. To detect the wavelength shift of the resonant transmission inclination caused by the analysis medium put on the sensor surface, a transport-type infrared spectral sensor based on a graphene nanoribbon array was presented [193]. Dynamically tuned gated self-biased graphene band arrays selectively excite local SPR modes at different frequencies [194].

**Figure 12:**
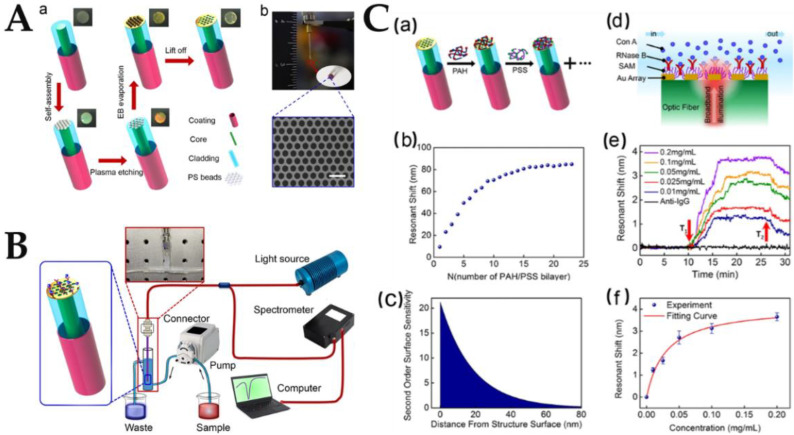
(**A**) Fabrication process diagram of fiber optic probe based on nanopore array. (**B**) Schematic diagram of experimental setup for sensing performance of fiber nanoprobes. (**C**)(a) Diagram showing the fiber tip takes in alternating layers of PAH and PSS; (**C**)(b) Shift of the resonant dip angle’s wavelength as the number of PAHs/PSS bilayers increase; (**C**)(c) The second-order surface sensitivity curve of the angle of inclination of the resonance; (**C**)(d) Side view of Con A and RNase B bound to a flow channel on a gold hole array; (**C**)(e) Resonance shifts in real-time for different Con A dip angles; and (**C**)(f) Resonance changes when Con A specifically binds to RNase B at different concentrations of fiber sensing probes. Reprinted with permission from Scientific Reports, Copyright 2019, Springer Nature [190].

Liang et al. developed a low-cost, high-growth multimode fiber optic biosensor integrated with plasma nanopore arrays using self-assembled nanospheres photolithography [190]. Large facet areas and high numerical apertures of MMF increased the incident light coupling effectiveness. The plasma fiber nanoprobes feature a clear reflectance inclination and a potent near-field electromagnetic amplification as a result of the resonance coupling of the plasma mode. The sensor performance of the plasma fiber optic nanoprobe was investigated, and its use in real-time tracking of protein molecule binding was further shown. Figure 12A summarizes the preparation process of nanopatterned fiber nanoprobes using self-assembled nanosphere lithography and the SEM images of the prepared Au nanopore arrays. The experimental configuration of the sensing performance test of the nanopatterned fiber probe is shown in Figure 12B. Figure 12C(a) illustrates the assembly of poly allylamine hydrochloride (PAH) and poly styrene sulfonate (PSS) on a fiber optic probe. Figure 12C(b) shows that the response wavelength of the resonant dip angle clearly shifts to red with increasing PAH/PSS bilayer. The findings demonstrate that the resonant angle has both a strong surface electric field location and great surface sensitivity. The second-order surface sensitivity of the resonant angle is shown in Figure 12C(c). It can be observed that the second-order surface sensitivity of the resonant mode decreases rapidly with the distance from the nanostructure surface. As shown in Figure 12C(d), concanavalin A (Con A) has been employed to selectively bind ribonuclease B (RNase B) on a monolayer (11-mercaptoundecanoic acid) for protein sensitivity assessment in order to further illustrate the sensing capabilities of plasma fiber nanoprobes as unlabeled biosensors. As shown in Figure 12C(e), the sensor graph was obtained by monitoring the wavelength position change of resonance inclination λ. The effect of the longest offset on protein Con A concentration is seen in Figure 12C(f). It is obvious that the protein concentration of Con A in the initial area is inversely correlated with the wavelength shift. The majority of RNase B sites were filled by protein Con A as its concentration rose, reaching wavelength shift saturation. The outcomes of the experiments demonstrate that the nanopore array multi-mode fiber probe is the best option for creating portable and miniature biosensor systems.

## 6. Future Prospects

In several nanomaterials, the particle size is smaller than the wavelength of the composed electron because their size and dielectric constant are close to the size and dielectric constant of small biological substances, so they are favored in the development of biosensors. Nanomaterials with good biocompatibility can not only detect ordinary biological substances but also be used in imaging studies to test the efficacy of emerging microscopic technologies for biomolecular tracking. It presents superior properties that conventional materials do not have. According to the research trend of other biosensor platforms, it is believed that in the future, QDs, nanocomposite materials, nano-diamond, and other nanomaterials will also enter the development field of optical fiber biosensors.

At present, optical fiber sensing technology has made corresponding progress in DNA, bacteria, cancer cells, viruses, glucose, and other biomedical fields. In the context of the global epidemic of coronavirus disease 2019 (COVID-19), the use of optical fiber biosensing technology to innovate the existing diagnostic detection technology is expected to achieve subversive innovation results and achieve clinical application. However, nanomaterial-based fiber optic biosensors still have shortcomings. Nanomaterials for biosensing should have high biocompatibility and low toxicity. Therefore, optical fiber biosensing probes based on nanomaterials are more suitable for in vitro detection rather than in vivo detection. Long-term use in vivo testing may retain long-term deleterious effects on the body. This also means that healthy non-toxic nanomaterials have long-term research significance in the field of biosensing applications.

Nanomaterials’ ultrathin nature is a huge benefit for sensing applications. A growing variety of nanomaterial-based sensors have recently shown great sensitivity. Nanomaterials often have a large surface area due to their extreme thinness. Therefore, nanomaterials not only enable smaller sensors but also provide significantly greater detecting surface areas. More “sensing points” on a larger sensing surface area will increase the biosensor’s sensitivity by increasing its overall sensitivity. Nanomaterials can, therefore, greatly enhance the sensing capabilities of sensors. Nanomaterials generally have a number of benefits that can aid researchers in designing highly sensitive sensors, including large surface areas, high thermal conductivity, electrical conductivity, and charge-transfer capabilities. The substances to be tested can be precisely adsorbable or captured by the right receptors for biological substance detection, which can also trigger related biochemical reactions. As a result, choosing the right receptors and their ability to fix onto targets are crucial steps in increasing the specificity and sensitivity of sensors.

## 7. Conclusions

In this work, the latest research progress of various nanomaterials in the development of fiber optic biosensors was discussed. The binding of nanomaterials to biological receptors effectively improves the sensitivity, LoD, specificity, and response time of the sensing probe, providing multiple benefits for the performance of fiber optic biosensors. The development of fiber optic biosensors is facilitated by the knowledge of the nanomaterial interactions in these recognition systems, such as the size-dependent signal amplification of nanoparticles, plasma resonance, and charge transfer capabilities. The versatility and affordability of detection are enhanced by the compatibility of nanomaterials with optical detection platforms. Simple and portable biosensors can timely assess the concentration index of human biological substances to achieve early prevention, early detection, and early treatment to effectively reduce the incidence of related diseases. Recently, the incorporation of various nanomaterials into multifunctional nanocomposites has also become more popular. This trend enhances the platform’s signal-to-noise ratio while also increasing sensitivity, making it ideal for commercialization.

## Figures and Tables

**Table 1:** Comparison of different fiber optic sensing probes modified with QDs.

Optical Fiber Structure	Sensing Mechanism	Nanomaterials	Analyte Biomolecule	LoD	Ref.
Dual-arm fiber optics	Fluorescence	ZnCdSe QDs	Pesticide	2.5 μg/L	[35]
Tapered MMF	Fluorescence	QDs	Staphylococcus aureus	n.r. ^a^	[37]
Large-core quartz optical fibers	Fluorescence	CQDs	Glucose	6.43 μΜ	[56]
				25.79 nM	
Large diameter quartz fiber	Fluorescence	N-CQDs	Acetyl choline	162.8 μΜ	[66]
				2.9 nM	
Large diameter quartz fiber	Fluorescence	CQDs	NO	9.12 nM	[73]
Exposed core fibers	Fluorescence	CdTe/CdS QDs	NO	0.01 nM	[74]
Tapered SMF	Fluorescence	CQDs	Dopamine	46.4 nM	[81]
Plastic fiber	Fluorescence	CdSe QDs	NO	0.1 nM	[87]
Plastic-clad multimode silica fiber (HPCF)	Fluorescence	CQDs-COD	Cholesterol	1 μΜ	[88]

^a^ not reported.

**Table 2:** Comparison of different fiber optic sensing probes modified with metal nanoparticles.

Optical Fiber Structure	Sensing Mechanism	Nanomaterials	Analyte Biomolecule	LoD	Ref.
Tapered SMF	LSPR	AuNPs	Cholesterol	53.1 nM	[18]
MMF-HCF-MMF	SPR	AuNPs	DNA	1 pM	[41]
Unclad MMF	LSPR	AuNPs	Taurine	53 μM	[42]
Ω-shaped	LSPR	AuNPs	MCF-7 cancer cells	12 cells/mL	[43]
Tapered SMF	LSPR	AuNPs	Glucose	322 μM	[57]
Tapered SMF	LSPR	AgNPs	Dopamine	0.058 μM	[63]
SMF-HCF	LSPR	AuNPs	Cholesterol	25.5 nM	[75]
Tapered SMF	LSPR	AuNPs	Uric acid	175 μM	[76]
SMSMS	LSPR	AgNPs/CuONPs	Uric acid	0.35 mM	[102]
				69.26 μM	
Ex-TFG	LSPR	AuNPs	Glucose	2.5 nM	[107]
Tapered SMF	LSPR	AuNPs	DNA-c	1.32 fM	[108]
MMF	FO-LSPR	AuNPs	Thyroglobulin	6.6 fg/mL	[109]
SFS	Chemical luminescence	AuNPs	Prostate-specific antigen	0.3 pg/mL	[110]
MMF	LSPR	AgNPs	Glycoprotein	30.76 nm/ppm	[111]
PSF	Coupling effect between SPR and LSPR	AuNPs	Human IgG	37 ng/mL	[112]
MMF	SERS	AgNPs	Antibiotics R6G	1 nM	[113]
SMS	LSPR	PVA-AgNPs	L-Cysteine	136.6 μM	[114]
Tapered SMS	LSPR	AgNPs	L-Cysteine	63.25 μM	[115]
SMPMS	LSPR	AgNPs	Collagen-IV	126.07 ng/mL	[116]

**Table 3:** Comparison of different fiber optic biosensor probes modified with metal film.

Optical Fiber Structure	Sensing Mechanism	Nanomaterials	Analyte Biomolecule	Sensitivity	Ref.
MMF-SMF-MMF	SPR	Au film	Glucose	3.10 pm/(mg/dL)	[5]
U-shape Fiber	SPR	Au film	Glucose	0.172 nm/(μg/mL)	[15]
			Sucrose	0.738 nm/(mg/mL)	
Tilted TFBG	SPR	Ag film	Glucose	0.5 dB/mM	[26]
Unclad MMF	SPR	Au film	CRP	1.17nm/lg (μg/mL)	[144]
Unclad fiber	SPR	Ag film	NS1 antigen	54.7nm/(μg/mL)	[145]
HPCF	SPR	Au film	Glucose	6.6 nm/mM	[146]
			Cholesterol	63 pm/nM	
PCS MMF	SPR	Cu film	BSA	1.907 nm/(mg/mL)	[147]
Unclad Fiber	SPR	Co/Ag film	Ammonium	0.131 nm/ppm	[148]

**Table 4:** Comparison of different fiber optic biosensor probes modified with GO.

Optical Fiber Structure	Sensing Mechanism	Nanomaterials	Analyte Biomolecule	Sensitivity	Ref.
SMF-PCF-SMF	SPR	GO	Glucose	0.1225 nm/(mg/mL)	[25]
Ex-TFG	Langmuir adsorption	Monolayer GO	Hemoglobin	3.83 nm/(mg/mL)	[28]
		Bilayer GO		4.33 nm/(mg/mL)	
		Three layers GO		8.21 nm/(mg/mL)	
LPG	Interferometer	GO	25-hydroxyvitamin D3	1.0 ng/mL	[31]
MMF-HSC-MMF	SPR	GO-AuNRs	Amino acids	n.r. ^a^	[40]
MMF-PCF-MMF	SPR	Au film/GO	Human IgG	0.3 nm/(μg/mL)	[151]
Tapered SMF	LSPR	AuNPs/GO	Glucose	1.06 nm/mM	[152]
MTLPG	Optical-tweezer effect	GO nanosheets	Hemoglobin in water	2 nm/(mg/mL)	[153]
			Hemoglobin in urea	1 nm/(mg/mL)	
			Hemoglobin in glucose	0.73 nm/(mg/mL)	
MMF-SMF	FRET	GO nanosheets	Dopamine	0.51 kHz/μM	[154]
			Nicotine	0.2 kHz/nM	
			ssDNA	8.8 kHz/nM	
LPFG	SPR	GO	Glucose	0.77 nm/(mg/mL)	[155]
TFG	Wavelength modulation	GO	Glucose	0.25 nm/mM	[156]
SMF-NCF-SMF	SPR	GO	Glucose	0.04 nm/(mg/mL)	[157]
PCS	SPR	GO/Ag film	Human IgG	0.4985 nm/(μg/mL)	[158]

^a^ not reported.

**Table 5:** Comparison of different fiber optic sensing probes modified with MoS_2_.

Optics Fiber Structure	Sensing Mechanism	Nanomaterials	Analyte Biomolecule	LoD	Ref.
SMF-MCF-MMF-SMF	LSPR	AuNPs/GO/MoS_2_	Creatinine	128.4 μM	[7]
D-shaped SMF	SPR	MoS_2_/GO	Glucose	n.r.^a^	[13]
Etched MPM	LSPR	GO/AuNPs/MoS_2_	Cardiac Troponin I	96.263 ng/mL	[91]
SMF-MCF	LSPR	AuNPs/MoS_2_	Shigella Bacteria	1.56 CFU/mL	[103]
MMF-Tapered MCF-MMF	LSPR	AuNPs/MoS_2_	Acetylcholine	14.28 μM	[163]
MMF-Etched MCF-MMF				71.30 μM	
Etched MMF	SPR	MoS_2_	*E. coli*	97 CFU/mL	[165]
Etched MMF	SPR	MoS_2_	BSA	0.29 μg/mL	[174]

^a^ not report.

**Table 6:** Comparison of different fiber optic sensing probes modified with MXene.

Optics Fiber Structure	Sensing Mechanism	Nanomaterials	Analyte Biomolecule	LoD	Ref.
CTC	LSPR	AuNPs/Nb_2_CTx MXene	Creatinine	86.12 μM	[16]
PTIF	SPR	Ti_3_C_2_-MXene	GDF11	0.55 pg/L	[176]
Tilted fiber Bragg grating	PTS	Nb_2_CTx MXene	Pesticide	0.35 ppm	[178]
Tapered SMF	SPR	Nb_2_CTx MXene	BOD	57 μg/mL	[182]
SMF-NCF-TNCF-NCF	SPR	Ti_3_CN MXene	NaCl	n.r. ^a^	[183]
PCS	SPR	Ti_3_C_2_-MXene	VOC	n.r. ^a^	[184]

^a^ not reported.

## Data Availability

Not applicable.
